# Global DNA Methylation and DNA Methyltransferase Status Among Cigarette Smokers in Saudi

**DOI:** 10.3390/life15020171

**Published:** 2025-01-24

**Authors:** Areej AlAmeer, Samar Sultan

**Affiliations:** 1 Medical Laboratory Sciences, Faculty of Applied Medical Sciences, King Abdulaziz University, Jeddah 80200, Saudi Arabia; aalamer0024@stu.kau.edu.sa; 2 Regenerative Medicine Unit, King Fahad Medical Research Centre, King Abdulaziz University, Jeddah 80200, Saudi Arabia

**Keywords:** epigenetic changes, cigarette smoke, DNA methylation, DNA methyltransferase

## Abstract

Smoking is a serious public health concern worldwide. It is a common environmental factor causing epigenetic alterations. This study aimed to explore the effect of smoking on DNA methylation by quantifying global DNA methylation, measuring the concentrations of plasma DNA methyltransferases (DNMT1, DNMT3A, and DNMT3B) among cigarette smokers in Saudi, and comparing these results with those of nonsmokers. Whole blood specimens were collected from Saudi cigarette smokers (n = 36) and non-smokers as controls (n = 36). Global DNA alteration was determined by a 5-methylation Cytosine (5-mC) colorimetric assay, and the concentration of DNMT proteins was measured by enzyme-linked immunosorbent assay (ELISA). DNA hypomethylation was found in smokers compared with controls (*p* < 0.001). Cigarette smokers showed significantly increased DNMT concentrations (DNM-1, DNMT-3A, and DNMT-3B) (*p* < 0.005). Global DNA hypomethylation correlated significantly with smoking duration (r = −0.854, *p* = 0.040) but not with other clinical parameters. In addition, DNMTs also were significantly correlated with smoking duration DNMT1 (r = 0.033, *p* = 0.002), DNMT3A (r = 0.431, *p* < 0.001), and DNMT3B (r = 0.553, *p* = 0.015). Our findings suggest that cigarette smoke induces epigenetic dysregulation, a principal player in cancer and various diseases through global DNA hypomethylation and high concentrations of DNMTs among cigarette smokers compared with nonsmokers.

## 1. Introduction

Smoking is a major public health risk, accounting for most global causes of morbidity, disability, and premature mortality [1]. It is a major risk factor for chronic diseases, such as cancer and cardiovascular disease [2,3]. A strong correlation exists between lung cancer and cigarette smoke, and cessation helps minimize the risk of lung cancer development [4]. In the Kingdom of Saudi Arabia, the number of smokers has increased dramatically over the past few decades, resulting in a significant number of deaths. Globally, Saudi Arabia ranks fourth in tobacco sales and imports and shows an increasing tobacco consumption among adults aged 15 years and above [5]. The tobacco consumption among adult cigarette smokers in 2019 was approximately 19.8%, with approximately 30.0% males and 4.2% females [6].

Cigarettes contain nearly 4000 chemical components, including 69 carcinogens, such as nitrosamines, polycyclic aromatic hydrocarbons, aromatic amines, hydrogen cyanide, formaldehyde, lead, arsenic, ammonia, polonium-210, and benzene [7]. Nicotine is an alkaloid extracted from tobacco. It is among the most harmful medications. Cigarettes contain a small amount of nicotine, around 10 to 12 mg, but smokers inhale approximately 1.1 to 1.8 mg from one cigarette [8].

Epigenetics is an important factor underlying the onset and progression of human diseases, such as cancer and respiratory disorders [9]. Three main epigenetic mechanisms exist, including DNA modification, DNA methylation, histone modification, and RNA-mediated processes (non-coding RNAs) [10]. DNA methylation is a biological process involving two alterations in which methyl groups (-CH3) are enzymatically added to or removed from the fifth carbon of cytosine or the sixth carbon of adenine in DNA [11]. The most common DNA methylation alteration is 5-methylcytosine (5 mC), which occurs in the cytosines preceding guanine nucleotides (CpG sites). DNA methylation can inhibit the binding of transcription factors to promoter regions, resulting in suppressed gene expression [11].

DNA methylation is regulated by a family of enzymes known as DNA methyltransferases enzymes (DNMTs) which catalyze the addition of a methyl group from S-adenosylmethionine to cytosine in DNA, forming 5 mC [12]. DNMTs maintain methylation patterns during DNA replication. Three families of these enzymes exist: DNMT1, DNMT3A, and DNMT3B. DNMT1, the first identified DNMT, is an epigenetic regulator that maintains the DNA methylation patterns during replication. DNMT3A and DNMT3B act as de novo methyltransferases, creating new DNA methylation patterns during embryogenesis and genomic imprints throughout germ cell development [13]. Previous studies have found an association between smoking and alterations in DNA methylation status, and this has been implicated in the occurrences of lung cancer [14], inflammatory illnesses [15,16], and heart disease [17]. However, the effects of cigarette smoking on healthy smokers without complications remain unclear. Another study suggests that smoking-related DNA methylation biomarkers are strongly linked to cognitive function, brain structure, physical health, and psychosocial well-being [9]. Another study reported changes in the levels of global methylation and hydroxymethylation in oral epithelial cells [18]. Moreover, studies on the effect of cigarettes on the Saudi population are limited, and samples were only collected from males because smoking among women in Saudi is a social taboo, resulting in challenges to collecting samples from females.

In this study, we aimed to explore the effect of smoking on DNA methylation by quantifying global DNA methylation, measuring the concentration of plasma DNA methyltransferase proteins (DNMT1, DNMT3A, and DNMT3B) among cigarette smokers from both males and females in Saudi and compared their results with those of non-smokers. The correlation between the smokers’ clinical parameters and smoking duration with DNA methylation status was also investigated.

## 2. Materials and Methods

### 2.1. Study Participants and Samples

This study adhered to the Declaration of Helsinki. Ethical approval was obtained from the Research Ethics Committee (IRB Committee) of the Directorate of Health Affairs, Makkah region (No.: H-02-K-076-1023-1007, date 30 October 2023). All participants provided written informed consent before inclusion in the study.

Participants were recruited from November 2023 to April 2024. Saudi cigarette smokers were recruited from the smoking clinic at the Ibn Sena Hospital in Makkah City, Saudi Arabia. Of the 100 available Saudi cigarette smokers, 36 were included: 21 male and 15 female smokers. Adult smokers aged 20–60 years were included in the study. Participants who reported consumption of other tobacco products (e.g., water pipes or electronic cigarettes), who had been smoking for less than a year, who did not smoke daily, had a chronic illness (e.g., cancer, hypertension, diabetes mellitus), heart disease, were under a smoking cessation routine, and those who had started taking cessation medication, were excluded.

In addition, a control group included 36 participants, 10 males and 26 females, who were randomly selected from consecutive individuals attending the other outpatients’ clinics with the following criteria: apparently healthy Saudi individuals aged 20–60 years and those who expressed their consent to participate in the study, free of chronic illness as in the case group. Simultaneously, family members of smokers were excluded due to the influence of passive smoking.

In brief, 2 mL peripheral blood samples extracted from male and female participants and collected in two ethylenediaminetetraacetic acid (EDTA)-containing tubes were used to quantify global DNA methylation and determine the levels of plasma DNA methyltransferases. In addition, 2 mL of fasting venous blood was collected in lithium heparin-containing tubes (green tops) from cigarette smokers, males and females, and analyzed for cholesterol, triglyceride, and fasting blood glucose levels using an automated Dimension^®^ EXL200 Clinical Chemistry System (Siemens, Munich, Germany).

### 2.2. Quantification of Global DNA Methylation

Total DNA was isolated from whole blood samples using a HiGene™ genomic DNA preparation kit (Biofact, Daejeon, Republic of Korea) following the manufacturer’s recommended protocol. The quality and integrity of the DNA were checked using a NanoDrop spectrophotometer (Thermo Fisher Scientific, Waltham, MA, USA). DNA samples with optical density (OD) 260/280 ratios between 1.8 and 2.1 were used.

The methylated DNA (colorimetric) 5-mC analysis was quantified using a 5-methylation cytosine colorimetric assay (Abcam, Cambridge, MA, USA). The assay was performed following the manufacturer’s instructions. In brief, 100 ng of pure DNA was treated with a binding solution and incubated at 37 °C. The plate was washed with buffer. DNA samples were incubated with anti-5-mC monoclonal and detection antibodies. After the addition of the enhancer and developer solutions, the absorbance was measured at 450 nm using a microplate spectrophotometer.

### 2.3. Determination of ELISA

The levels of plasma DNA methyltransferases DNMT1 (ELK3561; ELK Biotechnology, Denver, CO, USA), DNMT3A (ELK4571; ELK Biotechnology), and DNMT3B (ELK4719; ELK Biotechnology) in the samples were determined using ELISA kits following the manufacturer’s instructions. The OD at 450 nm was measured using a microplate spectrophotometer following the manufacturer’s instructions.

### 2.4. Statistical Analysis

Statistical analyses were performed using Statistical Package for Social Science (IBM SPSS, version 27) software, Armonk, NY, USA. Descriptive statistics were used to summarize the demographic, clinical, and laboratory findings. Qualitative variables were expressed as percentages, while numerical continuous variables were expressed as the median quartile range. The normality of quantitative variables was assessed using Shapiro–Wilk tests, and these did not follow a normal distribution. Non-parametric statistical tests were thus used. A power of 0.8 was chosen for the study, and the affected sample size was calculated with OpenEpi (Open-Source Epidemiologic Statistics for Public Health). The minimum sample size was determined by a two-sided confidence interval of 95%, the ratio of cases to controls of 1 to 1, the hypothetical proportion of controls with exposure of 40, the hypothetical proportion of cases with exposure of 72.48, and the odds ratio at 3.95. A paired sample *t*-test (two-tailed significance) was used to compare differences between study groups. The strength and direction of the linear relationships were assessed using Spearman’s correlation coefficient (r). Binary logistic regression analysis was used to identify significant variables related to methylation status in cigarette smokers. Statistical significance was set at *p* < 0.05.

## 3. Results

### 3.1. Socio-Demographic Data of the Participants

Table 1 details the baseline characteristics of the two groups. The cigarette smokers-cases group included 36 participants (male = 21, female = 15) and the median quartiles range of ages was 37.00 (32.25–39.75), male 35.0 (30.5–39.0), female 37.0 (33.0–44.0), while the control group included 36 (male = 10, female = 26) healthy subjects (non-smokers) and the median quartiles range of ages for the control group was 33.00 (26.25–36.50), male 34.0 (24.0–37.0), female 33.0 (31.0–35.0). In the smoker’s group, the average duration of smoking was 15 years (7–20), depending on smoking duration was divided into three groups: 36.1% smoked from 1 to 10 years, 41.7% smoked from 11 to 20 years, and 22.2% smoked more than 20 years.

**Figure 1:**
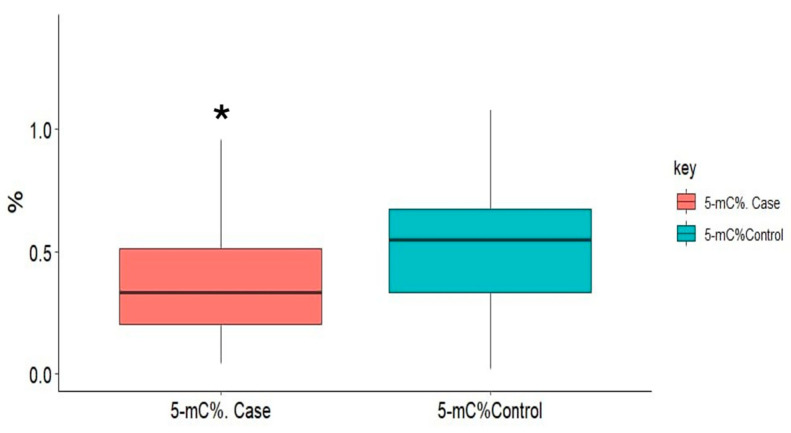
Alterations in global DNA methylation level in cases (cigarette smokers) compared to controls (nonsmokers). * Statistically significant at *p* < 0.05.

DNA methylation quantification in smokers compared with controls showed a significant decrease in the 5-mC% status (*p* < 0.001) in cigarette smokers compared with the control group (Figure 1, Table 2).

### 3.2. Expression of DNMTs in Cigarette Smokers

**Figure 2:**
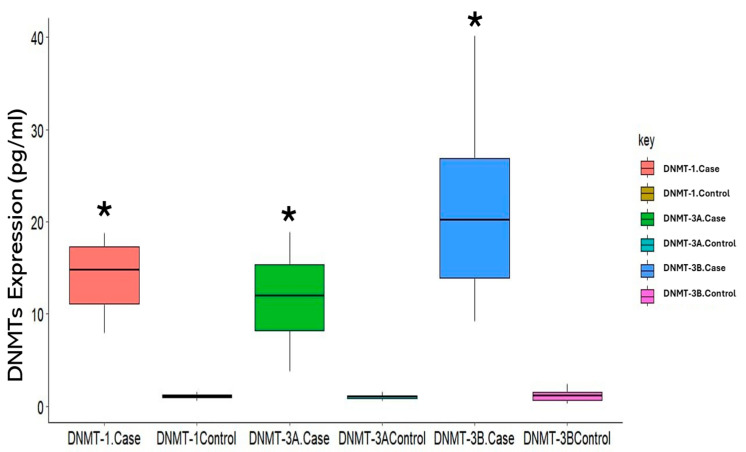
The expression levels of DNA methyltransferases: DNMT1, DNMT3A, and DNMT3B (pg/mL) between cases (cigarette smokers) and controls (nonsmokers). * Statistically significant at *p* < 0.05.

Expression levels of DNMTs between smokers’ groups and healthy controls are shown in Table 2. Plasma concentrations of DNMT1, DNMT3A, and DNMT3B were significantly higher in cigarette smokers versus nonsmoker controls (*p* < 0.0001, *p* = 0.003, and *p* = 0.012, respectively) (Figure 2, Table 2).

### 3.3. Association of Clinical Parameters with DNA Methylation Status

We performed a Spearman’s correlation analysis in the smokers’ group with DNMT1, DNMT3A, DNMT3B, methylation status, and age; the measured biochemical parameters revealed a non-significant correlation (Table 3).

**Figure 3:**
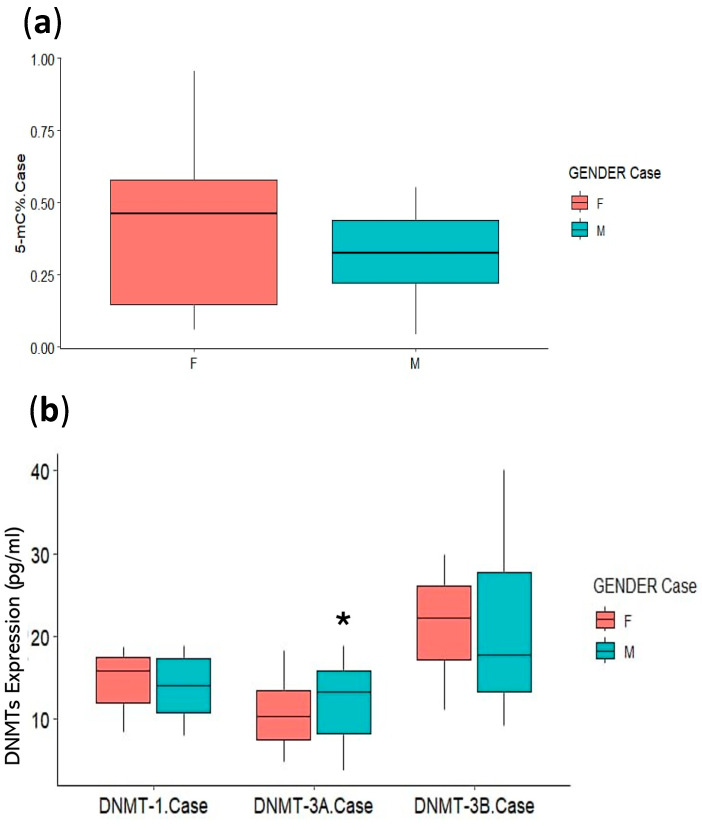
A binary logistic regression between smokers’ gender and (**a**) % 5 mC, and (**b**) DNMTs. * Statistically significant at *p* < 0.05.

A binary logistic regression (Table 4) was performed between smokers’ gender and DNMT levels and methylation status, and a non-significant association was found for all except between gender and DNMT3A Exp(B) with DNMT3A (1.529, *p* = 0.004) (Figure 3a,b).

### 3.4. Association of Smoking Duration with DNA Methylation Status

**Figure 4:**
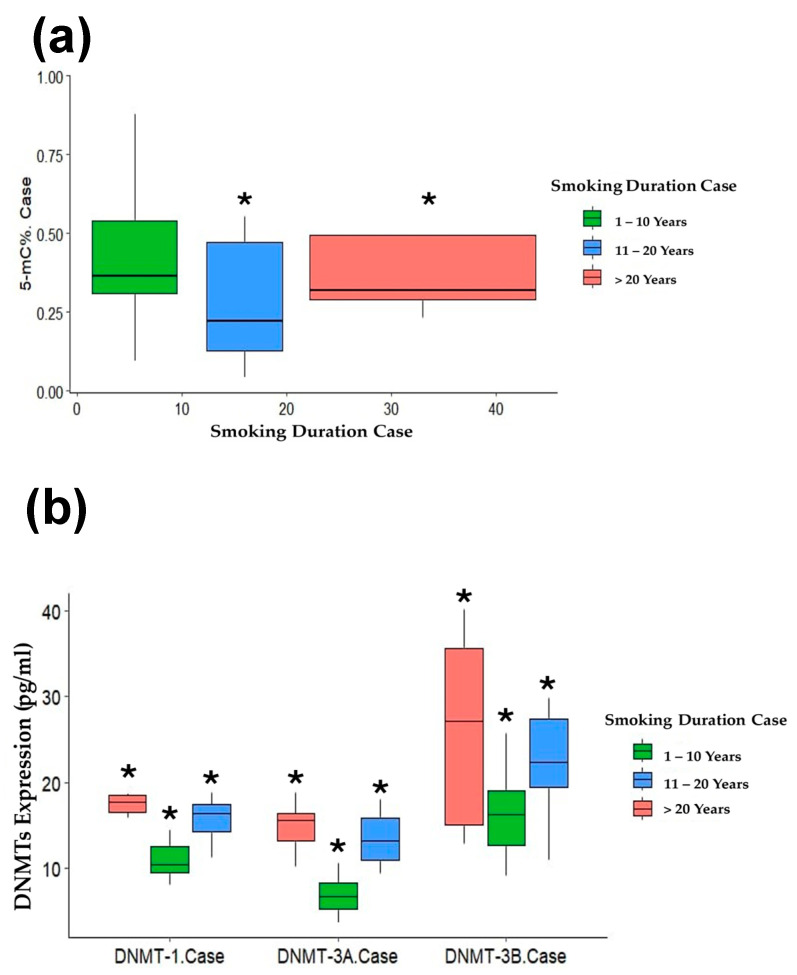
Spearman’s correlation of smoking duration with (**a**) % 5 mC, and (**b**) DNMTs. * Statistically significant at *p* < 0.05.

The smokers’ group, based on smoking duration, was divided into three groups: 1–10, 11–20, and >20 years. A correlation coefficient test between smoking duration in three groups and 5-mC% showed a significant correlation in smokers who smoked from 11 to 20 years and > 20 years (r = −0.203, *p* = 0.031, r = −0.854, *p* = 0.040, respectively) (Figure 4a, Table 5).

DNMTs were also significantly correlated with smoking duration in the three groups of smokers who smoked from 1–10 years, 11–20 years, and >20 years as follows: DNMT1 (r = 0.085, *p* = 0.001, r = 0.319, *p* = 0.002, r = 0.033, *p* = 0.002, respectively), DNMT3A (r = 0.124, *p* = 0.001, r = 0.361, *p* = 0.028, r = 0.431, *p* < 0.001, respectively), and DNMT3B (r = 0.148, *p* = 0.049, r = 0.177, *p* = 0.021, r = 0.553, *p* < 0.015, respectively), (Figure 4b, Table 5).

## 4. Discussion

Several studies have evaluated the association of tobacco smoke and its compounds with several epigenetic markers and blood DNA methylation [19]. In this study, we found global hypomethylation and increased concentrations of plasma DNA methyltransferases (DNMT1, DNMT3A, and DNMT3B) among cigarette smokers in Saudi compared with non-smokers, which may reflect the molecular mechanisms linking smoking and diseases, such as cancer.

A study comparing the data of smokers and non-smokers was conducted on a genome-wide methylation landscape of differentially methylated sites linked to smoking [7]. The results showed that smoking was associated with both hypo- and hyper-different methylation regions in these genes, with hypo-different methylation regions associated specifically with addiction and cancer [7]. Global DNA methylation showed extremely significant hypomethylation in the smoker group compared with that in the control group. This is consistent with a study by Mukhopadhyay et al., which showed that low global DNA methylation enhanced the proteasomal-mediated degradation of DNMT1 and DNMT3A in branchial arch-derived cells exposed to 80 μg/mL cigarette smoke for 24 hrs [20]. Interestingly, cigarette smoke induces pulmonary carcinogenesis through epigenetic alterations in methylation and its regulatory enzymes involved in DNA hypomethylation, decreases DNMT1, and increases DNMT3B protein expression in cultured respiratory epithelia [21]. Alterations in global methylation in cigarette smokers may mediate various diseases and cancers by altering gene expression patterns. Therefore, a correction in the DNA methylation status may pave the way for new procedures for preventing and treating cancers. Moreover, the molecular mechanisms underlying the change in DNA methylation levels that may affect smokers’ health are not yet fully understood. However, a study showed that hypomethylation may repress the population fighter genes, the aryl hydrocarbon receptor (AHR), in turn leading to the accumulation of environmental pollutants and subsequently increasing the risk of cancers among smokers [22]. Chemicals present in cigarette smoke, such as nitrosamine, can inhibit DNA methylation by regulating DNMT1 expression or DNA binding factor expression and activity. Modulation of DNA binding factor expression attached to GC-rich motifs in promoters inhibits DNA methylation. It can also damage the DNA strands, leading to the accumulation of DNMTs for repair [22]. Thus, smoking causes extensive accumulation of DNMTs in the nucleus [23,24] and may indirectly regulate DNMT expression [25].

Smoking causes extensive accumulation of DNMTs in the nucleus [23,24] and may indirectly regulate DNMT expression [25]. In this study, DNMT1 levels were higher among cigarette smokers compared with non-smokers. DNMT1 is a maintenance enzyme that plays a key role in the DNA methylation process that methylates DNA [26]. A growing body of evidence suggests that aberrant methylation of tumor suppressor genes is mediated by overexpression of DNMT1 [27,28]. Tennis et al. showed that overexpression of DNMT1 induced methylation of the tumor suppressor Wnt7a in non-small cell lung cancer, rendering it inactive and knocking down DNMT1 by shRNA, and activated it through decreasing its methylation in non-transformed lung epithelial cell lines cultured in 1% cigarette smoke condensate [29]. The authors suggested that therapeutically targeting DNMT1 could help demethylate and restore Wnt7a expression [29]. Wang et al. showed that the tobacco component nitrosamine 4-(methylnitrosamino)-1-(3-pyridyl)-1-butanone (NNK) induced the expression of DNMT1 protein and its stabilization in laryngeal cancer tissues, suggesting its role in the malignant progression of the larynx [23]. Cigarette smoking affects the expression DNMT1, which may be involved in cancer pathogenesis through de novo methylation of tumor suppressor genes.

In this study, we found an increased expression of DNMT3A in cigarette smokers compared to controls. Alterations in DNMT3A levels are associated with chronic diseases and cancers in cigarette smokers and non-smokers. Zhang et al. [15] suggest that epigenetic dysregulation of DNMT3a may enhance the harmful effects of smoking on the pathogenesis of inflammatory bowel disease. Dendritic cells (DCs) play a key role in chronic obstructive pulmonary disease progression, a type of chronic respiratory disease (COPD) [30]. Interestingly, DNMT3A overexpression has been reported during DC differentiation [31], and cigarette smoking promotes its increased expression in these cells, resulting in an imbalance and induction of T helper 17 (Th17) and regulatory T (Treg) cells in COPD [32]. Husni et al. showed that DNMT3A expression serves as an independent prognostic marker for lung adenocarcinoma and that strong expression of DNMT3A in tumors indicates a good prognosis. In contrast, a lack of expression induces tumor progression [33]. Thus, DNMT3A dysregulation in cigarette smokers may be involved in the initiation of lung and colon cancer pathogenesis.

Individuals with cigarette smoking habits are at higher risk of developing lung cancer, which is the leading cause of mortality worldwide [34]. The quantification of the reversibility of smoking-induced methylation changes indicates that increasing the duration of smoking causes an increase in hypomethylation, and it can be reversible after stopping smoking [35]. These results align with our findings, showing a statistically significant negative correlation between smoking duration and methylation, indicating that hypomethylation increases with tobacco exposure and longer smoking duration.

Interestingly, DNMT3B shows increased expression while miRNA 29b mRNA expression is low in the whole blood of patients with lung cancer compared to healthy controls [36]. The authors concluded that cigarette smokers with increased DNMT3b and low miRNA 29b expression are at a higher risk of developing lung cancer than non-smokers with lower DNMT3B and higher microRNA-29b [36]. Huang et al. showed that the DNMT3B-149 TT genotype, which has increased promoter activity, along with DNA damage itself, can increase the risk of lung cancer caused by smoking [37]. Overexpression of splicing alterations in DNMT3B causes chromosomal instability and DNA hypomethylation in pericentromeric satellite regions of precancerous hepatic tissue and plays a key role in human hepatocarcinogenesis [38]. Together, these findings are consistent with our results that the smoker group had a significantly higher level of DNMT3B than the control group, which could explain the consequences arising from its increased expression.

The main limitations of this study are the small sample size and the absence of long-term follow-up or data on other potential confounders (e.g., alcohol consumption, diet, and exercise). However, this study has several strengths. It provides an exploration of the significance of methylation status in smokers, which helps to establish a strong foundation for future investigations focused on enhancing our understanding of the different disease mechanisms and the early detection of cancers. Investigating methylation changes post-cessation or exploring gender-specific effects in the future is expected to provide a more comprehensive view.

## 5. Conclusions

Cigarette smoking induces epigenetic changes through global DNA hypomethylation and elevated concentrations of DNMT proteins, which may explain why cigarette smokers are at a higher risk of developing cancers and various other diseases than nonsmokers. Thus, DNA methylation status or DNMT levels might have great potential as biomarkers for early detection of smoking-related diseases.

## Figures and Tables

**Table 1:** Socio-demographic data of our control group (nonsmokers) and case group (smokers).

	Nonsmokers (Control) n = 36		Smokers (Case) n = 36	
**Personal Characteristics**	**No.**	**%**	**No.**	**%**
**Age (years)** **(Median-25th-75th quartiles)**	33.00 (26.25–36.50)		37.00 (32.25–39.75)	
**Gender**				
Male	10	27.8	21	58.3
Female	26	72.2	15	41.7
**Nationality**	Saudi individuals only			
**Duration of smoking (years)**			15 (7–20)	
1 to 10 years			13	36.1
11–20 years			15	41.7
>20 years			8	22.2
**Smoking type**			Cigarettes only, excluding water pipes or electronic cigarettes	
**Laboratory parameters**				
Glucose (mg/dL)			97.4 (88.7–100.7)	
Cholesterol (mg/dL)			183.5 (177.1–200.5)	
Triglyceride (mg/dL)			138.9 (129–150.4)	

**Table 2:** Our main findings of methylation status and DMNssTs of non-smokers (controls) and smokers (cases).

Parameters	Nonsmokers (n = 36)		Smokers (n = 36)		(95% CI)	*p*-Value
	Median	25–75 Quartiles	Median	25–75 Quartiles		
% 5 mC	0.549	(0.301–0.699)	0.331	(0.192–0.506)	(0.314–0.068)	<0.001 *
DMT1 (pg/mL)	7.433	(6.383–8.339)	15.445	(12.421–17.483)	(11.96–14.47)	<0.001 *
DNMT3A (pg/mL)	6.827	(6.100–8.151)	10.412	(7.861–14.606)	(9.34–11.99)	0.003 *
DNMT3B (pg/mL)	15.244	(10.647–20.465)	20.630	(14.429–27.433)	(17.52–23.52)	0.012 *

The *p*-value was obtained by paired samples *t*-test (two-tailed significance).* statistically significant at *p* < 0.05.

**Table 3:** Spearman’s correlation coefficient (r) between analysis between age and measured biochemical parameters with methylation status and DNMTs in cigarettes smokers.

Variable	% 5 mC			DNMT1			DNMT3A			DNMT3B		
	r-Value	(95% CI)	*p*-Value	r-Value	(95% CI)	*p*-Value	r-Value	(95% CI)	*p*-Value	r-Value	(95% CI)	*p*-Value
Age	0.149	(−0.373−0.301)	0.384	0.108	(0.156–0.696)	0.530	0.103	(0.042–0.632)	0.552	−0.067	(0.227–0.72)	0.699
Glu (mg/dL)	−0.282	(−0.566 −0.061)	0.095	0.246	(−0.100–0.539)	0.148	0.290	(−0.052–0.572)	0.086	0.046	(−0.296–0.377)	0.791
Chol (mg/dL)	0.120	(−0.227–0.440)	0.485	0.232	(−0.115–0.528)	0.174	0.163	(−0.184–0.475)	0.342	0.070	(−0.274–0.398)	0.687
TG (mg/dL)	0.034	(−0.307–0.367)	0.843	−0.018	(−0.353−0.321)	0.916	0.051	(−0.292–0.382)	0.768	−0.012	(−0.348–0.327)	0.945

Abbreviations: Glu: Glucose; Chol: Cholesterol; TG: Triglyceride.

**Table 4:** Binary logistic regression analysis of smokers’ gender related to methylation status and DNMTs.

Gender (Smokers) ^a^	*p*-Value	Exp(B)	95% CI for Exp(B)	
			Lower Bound	Upper Bound
Intercept	0.140			
% 5 mC	0.058	75.838	0.868	6628.110
DNMT1	0.162	0.791	0.570	1.098
DNMT3A	0.004 †	1.529	1.149	2.035
DNMT3B	0.997	1.000	0.874	1.144

† Statistically significant (*p* < 0.05); a, The reference category is Male; The dependent variable: Gender, Covariance: Methylation.

**Table 5:** Spearman’s correlation coefficient (r) between smoking duration with methylation status and DNMTs.

Parameters	1–10 Years n = 13		11–20 Years n = 15		>20 Years n = 8	
	r-Value	*p*-Value	r-Value	*p*-Value	r-Value	*p*-Value
% 5 mC	−0.084	0.101	−0.203	0.031*	−0.854	0.040 *
DMT1	0.085	0.001 *	0.319	0.002*	0.033	0.002 *
DNMT3A	0.124	0.001 *	0.361	0.028*	0.431	<0.001 *
DNMT3B	0.148	0.049 *	0.177	0.021*	0.553	0.015 *

* Statistically significant at *p* < 0.05.

## Data Availability

All data generated or analyzed during this study are included in the published article.
